# Bioactive Peptides from Germinated Soybean with Anti-Diabetic Potential by Inhibition of Dipeptidyl Peptidase-IV, α-Amylase, and α-Glucosidase Enzymes

**DOI:** 10.3390/ijms19102883

**Published:** 2018-09-22

**Authors:** Marcela González-Montoya, Blanca Hernández-Ledesma, Rosalva Mora-Escobedo, Cristina Martínez-Villaluenga

**Affiliations:** 1 Escuela Nacional de Ciencias Biológicas-Instituto Politécnico Nacional. Campus Zacatenco, Unidad Profesional “Adolfo López Mateos”, Calle Wilfrido Massieu esquina Cda. Manuel Stampa. C.P, Ciudad de México 07738, Mexico; marceglezmon@gmail.com (M.G.-M.); rosalmorae@gmail.com (R.M.-E.); 2 Instituto de Investigación en Ciencias de la Alimentación (CIAL, CSIC-UAM, CEI UAM+CSIC), Nicolás Cabrera 9, 28049 Madrid, Spain; b.hernandez@csic.es; 3 Institute of Food Science, Technology and Nutrition (ICTAN-CSIC), Juan de la Cierva 3, 28006 Madrid, Spain

**Keywords:** germinated soybean, gastrointestinal digestion, peptides, inhibitors, dipeptidyl peptidase, α-amylase, α-glucosidase

## Abstract

Functional foods containing peptides offer the possibility to modulate the absorption of sugars and insulin levels to prevent diabetes. This study investigates the potential of germinated soybean peptides to modulate postprandial glycaemic response through inhibition of dipeptidyl peptidase IV (DPP-IV), salivary α-amylase, and intestinal α-glucosidases. A protein isolate from soybean sprouts was digested by pepsin and pancreatin. Protein digest and peptide fractions obtained by ultrafiltration (<5, 5–10 and >10 kDa) and subsequent semipreparative reverse phase liquid chromatography (F1, F2, F3, and F4) were screened for in vitro inhibition of DPP-IV, α-amylase, maltase, and sucrase activities. Protein digest inhibited DPP-IV (IC_50_ = 1.49 mg/mL), α-amylase (IC_50_ = 1.70 mg/mL), maltase, and sucrase activities of α-glucosidases (IC_50_ = 3.73 and 2.90 mg/mL, respectively). Peptides of 5–10 and >10 kDa were more effective at inhibiting DPP-IV (IC_50_ = 0.91 and 1.18 mg/mL, respectively), while peptides of 5–10 and <5 kDa showed a higher potency to inhibit α-amylase and α-glucosidases. Peptides in F1, F2, and F3 were mainly fragments from β-conglycinin, glycinin, and P34 thiol protease. The analysis of structural features of peptides in F1–F3 allowed the tentative identification of potential antidiabetic peptides. Germinated soybean protein showed a promising potential to be used as a nutraceutical or functional ingredient for diabetes prevention.

## 1. Introduction

Diabetes mellitus (DM) is a metabolic disorder considered as one of the major health problems worldwide. It has been increasing constantly at 336 million people in the world and is projected to be present in 552 million people by 2030 [1]. Type 2 diabetes mellitus (T2DM) is characterized by dysregulation of carbohydrate, lipid, and protein metabolism, and results from impaired insulin secretion, insulin resistance, or a combination of both. It has been reported that 90% of all DM cases are T2DM [2]. After food consumption, insulin secretion is stimulated resulting from the combined effects of Glucose-Dependent Insulinotropic Polypeptide (GIP) and Glucagon-Like Peptide-1 (GLP-1) on the pancreatic β-cells [3]. These incretins hormones exert their metabolic effects through activation of their receptors. GLP-1 exerts important insulinotropic and glucagonostatic effects that can normalize blood glucose levels in patients with T2DM [4]. However, the biological activity of incretins is significantly reduced upon degradation by the action of dipeptidyl peptidase IV (DPP-IV), a ubiquitous serine protease whose inhibition is being considered a novel therapeutic strategy for managing T2DM [4,5]. Natural alternatives to DPP-IV inhibitory compounds have been identified including DPP-IV inhibitory peptide fragments which can be released following the hydrolysis of food proteins [6]. In addition, strategies which block carbohydrate hydrolyzing enzymes such as α-amylase and α-glucosidase have been used for the control of glucose homeostasis in diabetic patients. It is well known that blocking hydrolysis of complex starches to oligosaccharides in the small intestine and decreasing the rate of digestion of carbohydrates result in less glucose absorbed and transported in blood [7]. Additionally, α-glucosidase inhibitors are commonly used as oral hypoglycemic drugs. The guideline of the American Diabetes Association and the European Association for the Study of Diabetes have recommended the use of these inhibitors as a potential first-line agent or in combination with other anti-hyperglycemic drugs [8].

Food bioactive peptides have been described as potential therapeutic agents because of their wide range of preventive effects against chronic diseases. Generally, these peptides are encrypted in food proteins but can be released by hydrolysis from original protein during their gastrointestinal transit or by fermentation and/or germination processing [9]. Germination has been used as a strategy to enhance the nutritional value, phytochemical composition, and chemopreventive properties of soybean. Previous studies have shown an increase in bioactive compounds of soybean under optimal germination conditions [10]. It has also been reported that gastrointestinal digestion could aid the obtainment of bioactive peptides which potentially can exert multiple biological activities [11]. Recently, we have demonstrated that peptide fractions from gastrointestinal digestion of germinated soybean proteins show potent anti-proliferative effects on breast, cervix, and colon cancer cells [9,12]. However, no data on the anti-diabetic effects of these fractions are still available. Therefore, the aims of this study were to obtain bioactive peptides from germinated soybean protein isolate under conditions simulating gastrointestinal digestion and to characterize their in vitro anti-diabetic properties as inhibitors of DPP-IV, α-amylase, and α-glucosidase enzymes. Finally, the peptide sequences potentially responsible for the observed effects were identified.

## 2. Results and Discussion

### 2.1. In Vitro Anti-Diabetic Activity of Germinated Soybean Protein Hydrolyzed Under Simulated Gastrointestinal Conditions 

**Figure 1:**
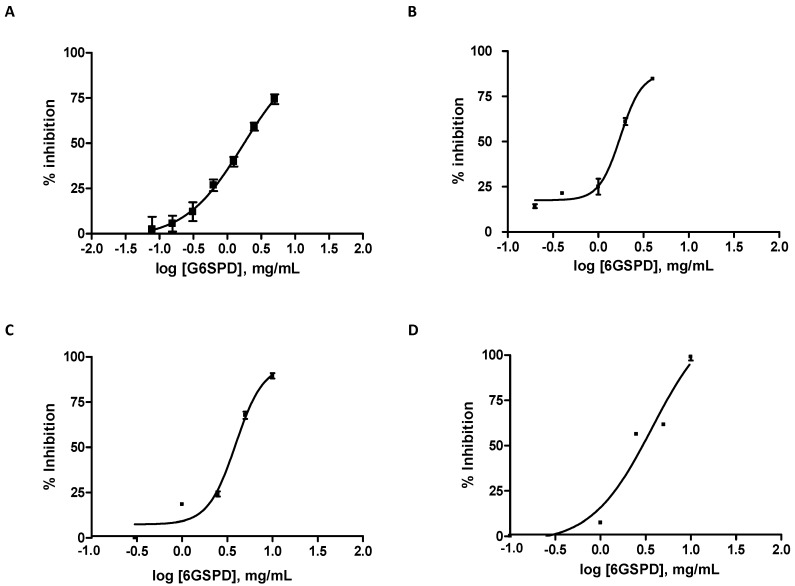
Germinated soybean protein digest inhibited DPP-IV (**A**), α-amylase (**B**) and maltase (**C**) and sucrase (**D**) activities of intestinal α-glucosidases Values are the mean ± standard deviation of three experiments performed in triplicate.

The gastroduodenal digest from the six days germinated soybean protein digest (6GSPD) and fractions obtained by ultrafiltration were analyzed by their ability to inhibit DPP-IV, α-amylase, and α-glucosidase. 6GSPD inhibited DPP-IV activity in a dose-dependent manner, see Figure 1A. After simulated gastrointestinal digestion, 6GSPD showed moderate DPP-IV inhibitory activity with an IC_50_ value of 1.49 ± 0.14 mg/mL that was ~10^3^ times less potent than Diprotin, in agreement with previous studies focused on protein derived hydrolysates; see Table 1 [7]. However, food and drug interventions may be complementary in the management of T2DM [13]. The IC_50_ value determined for 6GSPD was in the range described by Wang et al. [14] for cereal flours derived from highland barley, oat, and buckwheat and digested with pepsin and a trypsin-pancreatin mixture (IC_50_ = 0.99–3.91 mg/mL). Hydrolysates resulting from in vitro gastrointestinal digestion of other plant proteins such as hemp, pea, rice, and soy have also shown DPP-IV inhibitory activity (IC_50_ values between 1.85 ± 0.34 and 4.50 ± 0.55 mg of hydrolysate dry weight/mL) [15]. The main contributors to the DPP-IV inhibitory activity were ˃10 kDa and 5–10 kDa fractions that showed similar IC_50_ values of 1.18 ± 0.15 and 0.91 ± 0.17 mg/mL, respectively. This result is in agreement with previous studies that have described large peptides as important contributors to the DPP-IV inhibitory activity of food protein hydrolysates. Their DPP-IV substrate-like structural features enable them to competitively interfere with the combination of the synthetic substrate and DPP-IV [16]. Thus, a fraction containing whey protein-derived peptides in the 3–10 kDa range was found to be the most active inhibiting DPP-IV activity [17]. Similarly, three peptides comprising 13–15 amino acid residues and identified in tuna cooking juice hydrolysates showed potent DPP-IV inhibitory effects [18]. These authors suggested that the DPP-IV inhibitory activity could be dependent on the composition and sequence of amino acids, in addition to their length.

Starch is one of the main dietary sources of postprandial glucose in the blood after food intake. Salivary and pancreatic α-amylase and intestinal α-glucosidases are key enzymes for starch digestion in the gastrointesinal tract. Salivary and pancreatic α-amylase hydrolyse starch to produce maltose and other oligosaccharides by breaking the α-1,4 glycosidic bonds [19]. Small intestinal α-glucosidases, also known as maltase-glucoamylase (MGAM) and sucrase-isomaltase (SI), have two functional domains on the respective C- and N-terminal ends (Ct and Nt, respectively), that differ in substrate specificity [20]. Ct and Nt of MGAM have higher specificity for glucose oligomers and maltose, respectively, while Ct and Nt domains of SI have sucrase and isomaltase activities, respectively. A moderate α-amylase inhibition with a high α-glucosidase inhibition has been suggested as an effective strategy for decreasing dietary carbohydrates availability for glucose release in the gut [21]. Intestinal α-glucosidase inhibitors help to avoid hyperglycemia and maintain normal blood sugar levels. In this study, the inhibitory effects of 6GSPD on α-amylase, and maltase and sucrase activities of intestinal α-glucosidases were also examined, see Figure 1B–D; Table 1. 6GSPD was able to inhibit salival α-amylase as well as maltase and sucrase activities of intestinal α-glucosidases, although with a lower inhibitory potency (IC_50_ = 1.70, 3.73 and 2.90 mg/mL, respectively) compared to the positive control Acarbose (IC_50_ = 0.16, 0.07 and 0.03 mg/mL, respectively) (*p* ≤ 0.05). Drugs such as Acarbose act by inhibiting α-amylase and α-glucosidases and are used in the management of T2DM. Unfortunately, this drug is expensive which is a limitation to the therapeutic treatment of T2DM in low- and middle-income countries and there is evidence showing that long-term intervention with Acarbose results in gastrointestinal side effects (flatulence occurring in ≥1/10 patients, and diarrhoea and GI and abdominal pain in ≥1/100 to <1/10 patients) [22,23]. For these reasons, there is still a need for new therapeutic interventions. Other molecules such as peptides derived from food proteins have shown inhibitory effects towards α-amylase and α-glucosidases with IC_50_ values within the range of 0.19–23.30 mg/mL and 1.45–5.00 mg/mL, respectively [7,24,25,26]. Our results suggest that gastrointestinal digestion of 6GSP release potential peptide inhibitors of these enzymes which may have multiple positive health implications related to the control of the postprandrial glycemic response and most likely reduce appetite and food intake [27].

To identify the bioactive peptides responsible for the observed inhibitory effects of 6GSPD on glycohydrolysis, the different peptide fractions obtained by ultrafiltration were further screened for their α-amylase, sucrase, and maltase inhibitory activities, see Table 1. The peptide fraction with MW >10 kDa and <5 kDa showed higher α-amylase inhibitory activity (IC_50_ = 4.80 and 8.30 mg/mL) than 5–10 kDa (IC_50_ >10 mg/mL) fraction although a lower inhibitory potency in the formers was observed compared to 6GSPD (*p* ≤ 0.05). This finding suggests that peptides from different molecular weights might have synergistic or additive inhibitory effects. Regarding α-glucosidases, the peptide fractions 5–10 kDa and <5 kDa showed higher inhibitory potencies towards intestinal maltase (IC_50_ = 3.56 and 2.56 mg/mL, respectively) and sucrase (IC_50_ = 2.20 and 1.23 mg/mL) activities (*p* ≤ 0.05) than a peptide fraction > 10 kDa. Similar findings have been reported in earlier studies in which peptide fractions <5 kDa from quinoa [7], rice bran [28], and common bean [29,30] hydrolysates showed the strongest α-glucosidase inhibition. Interestingly, 6GSPD and their derived fractions (<5, 5–10 and >10 kDa) inhibited more effectively sucrase than maltase activity (*p* ≤ 0.05), which suggests a selective inhibition mode of germinated soybean-derived peptides. Consistently with our findings, Bautista-Exposito et al. [31] demonstrated that lentil hydrolysates and gastrointestinal digests are selective inhibitors of individual intestinal α-glucosidase subunits showing a higher inhibitory activity towards SI than MGAM.

### 2.2. Identification of Potential Anti-Diabetic Peptides

**Figure 2:**
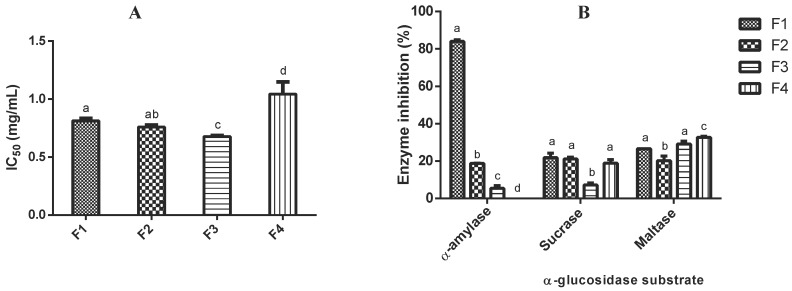
Dipeptidyl peptidase-IV (DPP-IV) (expressed as IC_50_ or peptide concentration needed to inhibit by 50% the original enzyme activity), α-amylase and α-glucosidases (expressed as % inhibition) inhibitory activities of peptide fractions collected by RP-HPLC from 5–10 kDa fraction obtained from germinated soybean protein digest (6GSPD). Peptide fractions (F1–F4) were tested at a dose of 1 mg/mL for α-amylase and α-glucosidases inhibitory assays. Values are the mean ± standard deviation. Different letters indicate significant differences among samples (*p* ≤ 0.05, Holm-Sidack’s test).

To identify the peptides potentially responsible for the enzyme inhibitory effects of 6GSPD, the peptide fraction of 5–10 kDa was selected and fractionated by semi-preparative RP-HPLC. Selection criteria were based on the greater potential of this fraction to inhibit DPP-IV and intestinal α-glucosidases, colon cancer cells proliferation, and inflammation in vitro [9]. To obtain a sufficient amount of purified peptides, chromatographic separations were performed repeatedly. Four fractions (F1–F4) were collected, lyophilized, and then used to determine their enzyme inhibitory activity (see Figure 2).

All fractions (F1–F4) showed inhibitory effects towards DPP-IV (IC_50_ values ranged from 0.81–1.04 mg/mL), although F3 (eluting from min 16 to 22) showed the lowest IC_50_ value, followed by F2 and F1. The IC_50_ values of these three fractions were lower than that determined in the 5–10 kDa peptide fraction (0.91 mg/mL), indicating that the purification process resulted in enrichment of peptides with potent DPP-IV inhibitory activity. On the other hand, to identify the most potent fractions to inhibit α-amylase and α-glucosidases (maltase and sucrase) inhibitory activities, F1 to F4 were screened at 1 mg/mL. F1 showed a strong inhibition of α-amylase at 1 mg/mL. Regarding α-glucosidase inhibitory activity, F1, F3, and F4 showed comparable maltase inhibitory activity (27%, 29%, and 33% inhibition, respectively) while F1, F2, and F3 showed similar sucrase inhibitory activity (~21% inhibition). 

Due to the fact that F1, F2, and F3 showed inhibitory activity towards DPP-IV, α-amylase, and α-glucosidases, they were selected and analysed by HPLC-MS/MS to identify the potential inhibitory peptides of these enzymes. The sequences, physicochemical properties (mass, net charge, and hydrophobicity) and biological potential of the main peptides present in the fractions F1, F2, and F3 from the 5–10 kDa fraction of 6GSPD are listed in Table 2. Fifteen, four, and seven peptides were detected in F1, F2, and F3 fractions, respectively. Peptides identified in all fractions were fragments of different length (6 to 18 amino acids) from β-conglycinin, glycinin, and P34 probable thiol protease. Hydrophobicity of the peptides from F1 ranged from +12.80 to +29.21 kcal/mol. For the peptides contained in F2, the range was wider (from +13.32 to +32.34 kcal/mol), and for those contained in F3 the range was narrower (from +14.43 to +26.14 kcal/mol). The more positive the value, the more hydrophobic the amino acids located in the peptide are. Table 2 also shows the potential biological function according to the BIOPEP database [32]. Most of the peptides identified in fractions F1, F2, and F3 contained encrypted in their amino acid sequence di- and tripeptides with antioxidant activity and potential to inhibit DPP-IV and angiotensin-converting enzyme.

To date, structure-activity relationships (SAR) for DPP-IV, α-amylase, and α-glucosidase are still unclear, although recent studies have provided important contributions to this research area. Regarding DPP-IV, SAR analysis, and sequential alignment of inhibitory peptides has demonstrated that the hydrophobicity of the amino acids at the Nt and next to the Ct positions of peptides played a role in their DPP-IV inhibitory properties. Other than hydrophobicity, aromatic amino acid residues of peptides are important to inhibit DPP-IV. A previous study [33] suggested that the potency of DPP-IV inhibitors is related to the presence of aromatic rings as they form hydrophobic interactions with the DPP-IV catalytic domain. More recently, it has confirmed the importance of the presence of hydrophobic residues and a proline at the first, second, third, or fourth Nt position of peptides displaying potent DPP-IV inhibitory properties [13,34]. Some of the peptides shown in Table 2 are consistent with previous studies. For instance, aromatic residues such as phenylalanine were found at the Ct end of peptides VVAEQAGEQGFE and HKNKNPF from F3. Proline was present at favorable positions of the N-terminal end of peptides VVNPDNNEN, EEPQQPQQ, QEPQESQQ, SQRPQDRHQ, and PETMQQQQQQ from F1 and SSPDIYNPQAGSVT from F3, although proline was not flanked by leucine, valine, phenylalanine, alanine, and glycine, in contrast with previous studies [35,36]. In addition, most of the peptides from F3 contained from four to six hydrophobic amino acid residues.

The mechanism through which peptides inhibit carbohydrate breakdown by α-amylase activity has been proposed recently. The interaction of amino acid residues of peptides to the α-amylase active site changes the position of the substrate by displacing it further away from the catalytic domain [37]. The amino acids composition of the peptides, as well as their sequences, play an important role in the inhibitory mechanism. In general, important interacting amino acids in α-amylase inhibitory peptides were found to be histidine, proline, and methionine in wheat and pinto bean [38]. A recent study has also demonstrated that hydrophobic (i.e., alanine, leucine, phenylalanine, valine, proline, and glycine) and hydrophilic amino acids (i.e., cysteine, histidine, and serine) were responsible for interacting with the active sites of human salivary α-amylase via hydrophobic and hydrogen bonds [24]. In this sense, many germinated soybean peptides identified in F1, F2, and F3 (fraction with the highest α-amylase inhibitory activity) contained hydrophobic and hydrophilic amino acid residues required for peptide binding to the α-amylase catalytic site. 

The SAR analysis of α-glucosidase inhibitory peptides, comprising of amino acid residues containing a hydroxyl group on their side chain (serine, threonine and tyrosine) at their Nt end, a proline residue closer to the Ct end, and alanine or methionine at the Ct position, showed that they are important for binding to or interacting with the α-glucosidase catalytic site [39]. Molecular docking studies have supported this structure–activity pattern. In this sense, inhibitory peptides bind mostly to the α-glucosidase catalytic domain by hydrogen bonds and electrostatic interactions [40,41]. Additionally, other structural features such as a peptide net charge of either 0 or +1, the presence of basic and sulphur-containing amino acids residues, and hydrogen bonding significantly enhanced the potency of α-glucosidase inhibitory peptides, whereas a net peptide negative charge reduces the inhibitory potency [40]. These structural features were found in peptides from the F1 (NAENNQRN, IKSQSES, GQSSRPQD, NLKSQQA, SQRPQDRHQ, QQQQQGGSQSQ, QQQQQGGSQSQKG) and F3 (RQNIGQNSSPDIYNPQAG) subfractions, see Table 2.

## 3. Materials and Methods

### 3.1. Materials

Soybeans (*Glycine max*) were obtained from Mexico City in a local market. Pepsin from porcine gastric mucosa (EC 3.4.23.1; ≥250 units/mg solid), pancreatin from porcine pancreas (EC 232-468-9; 8 × USP), the tripeptide IPI, called diprotin A, human recombinant DPP-IV enzyme, porcine pancreatic α-amylase (EC 3.2.1.1), and rat intestine α-glucosidase (EC 3.2.1.20) were purchased from Sigma-Aldrich (Madrid, Spain). The Quantitative Colorimetric Peptide Assay kit was from Pierce™ (Rockford, IL, USA), and the chromogenic substrate (H-Gly-Pro-*p*-nitroaniline) was purchased from Enzo Life Sciences Inc. (Farmingdale, NY, USA).

### 3.2. Preparation of Germinated Soybean Protein Isolate

Soybeans were germinated for six days according to a previous study [12]. Briefly, soybeans (100 g) were soaked in 600 mL distilled water containing 0.4 mL/L of colloidal silver for 2 h at 30 °C. Soaked seeds were placed in a germination chamber (China Weifang Kehua Machine Electricity, Shandong, China) at 30 °C for six days in darkness, and with an irrigation cycle of 10 s every 8 h. Germinated soybeans were dried in an oven (Shel Lab, Mexico DF, Mexico) at 40 °C for 24 h. The protein isolate from germinated soybean was prepared by alkaline extraction (pH 9.0) and isoelectric precipitation (pH 4.5). Phytochemicals, such as phenolic compounds, were extracted from protein isolates by alcoholic extraction following an optimized method [42]. The extraction procedure was repeated until phenolic compounds were not detected in alcoholic extracts. 

### 3.3. Simulation of Gastrointestinal Digestion

Soybean protein isolate was sequentially digested with porcine pepsin and pancreatin as previously described [12]. Briefly, protein isolate was dissolved in distilled water (5%, *w/v*) and its pH was adjusted to 2.0 with 1 N HCl (Merck, Darmstadt, Germany). Pepsin was added to reach an enzyme:substrate (E/S) ratio of 4% (*w/w*, protein basis) and the solution was incubated at 37 °C for 1 h. Then, pancreatin (E/S 4% *w/w*, protein basis) was added, and the pH was adjusted to 7.5 with 1 N NaOH (Merck). The solution was incubated at 37 °C for 2 h. The reaction was stopped by immersing the samples in a water bath at 100 °C for 10 min. 6GSPD was centrifuged at 16,000× *g* for 10 min, and the supernatant was collected and stored at −20 °C until further processing. Three peptide fractions (>10 kDa, 5–10 kDa, and <5 kDa) were obtained by ultrafiltration at 70 psi using 5 and 10 kDa cut-off hydrophilic membranes (Millipore, Billerica, MA, USA). All fractions were lyophilized and kept at −20 °C.

### 3.4. Measurement of the Enzymatic Inhibitory Activity

#### 3.4.1. DPP-IV Inhibitory Activity

DPP-IV inhibitory activity was measured following the protocol previously described [3]. Recombinant soluble human DPP-IV (0.26 mU per test well; 15 μL) was incubated in 96-well plates at 37 °C in the absence or presence of different concentrations (0.08–5 mg/mL) of samples (final volume 50 μL/well) for 10 min. The enzymatic reaction started after the addition of 50 μL of assay buffer containing H-Gly-Pro-*p*-nitroaniline to each well (final concentration 100 μM). Absorbance was read at 405 nm in a microplate reader (BMG Labtech Inc., Champigny-sur-Marne, France) at 2 min time intervals starting from 0 up to 30 min. Recorded data were plotted versus time. The best fit straight line was obtained in the time range in which the increase in the absorbance was linear. Then, data were expressed as % remaining activity in the presence of test samples versus control (without sample) and represented versus protein/peptide concentration. Diprotin A was used as a positive control (concentration range from 0.78 to 50 µM). Each sample was analysed three times. Data were plotted and fitted to logarithmic regression to obtain dose-response curves. The results were expressed as the IC_50_ value or peptide concentration needed to inhibit by 50% the original DPP-IV activity.

#### 3.4.2. α-Amylase Inhibitory Activity

The α-amylase inhibition assay was adapted from a previously described method [7]. Briefly, 50 µL of sample (concentration range from 0.2 to 4 mg/mL), positive control (acarbose used at a concentration range of 0.1–1.3 mg/mL), or negative control (distilled water) were added to 100 µL α-amylase solution (2 U/mL in 0.02 M sodium phosphate buffer pH 6.9). Test tubes were incubated at 20 °C in a Thermomixer orbital shaker (Eppendorf Iberica, Madrid, Spain) for 5 min. Later, 100 µL of 1% potato soluble starch solution (previously dissolved in 0.02 M sodium phosphate buffer pH 6.9 and boiled for 15 min) was added to each tube and incubated in a Thermomixer orbital shaker (Eppendorf Iberica) at 20 °C, 1000 rpm for 6 min. Finally, 100 mL of dinitrosalicylic acid solution was added, and the tubes were placed in a 100 °C water bath for 15 min. A volume of 800 mL of distilled water was added to the mixture, and the absorbance was read at 540 nm in a Synergy HT microplate reader (Biotek, Winooski, VT, USA). Percentage of inhibition was calculated relative to the negative control with 100% enzyme activity. IC_50_ values (concentration of sample in mg/mL that inhibits by 50% the original α-amylase activity) were calculated plotting the non-linear regression sigmoidal dose-response curves in GraphPad Prism 6.00 (GraphPad Software Inc., San Diego, CA, USA).

#### 3.4.3. α-Glucosidase Inhibitory

The α-glucosidase inhibitory activity was assessed by comparing the amount of glucose released by the α-glucosidase enzyme from its natural substrates, maltose and sucrose, in the presence and absence of hydrolysed samples. The α-glucosidase inhibition assay was performed following Vilcacundo et al. [7]. A quantity of 100 µL of samples (at a concentration range of 1–10 mg/mL), positive control (mM acarbose), or negative control (distilled water) were added to 50 μL of rat intestine α-glucosidase (1 U/mL in 0.1 M maleate buffer pH 6.9). Test tubes were incubated at 37 °C for 5 min. After pre-incubation, 50 μL of substrate (2 mM maltose or 20 mM sucrose) was added to each tube. The reaction mixtures were incubated using a Thermomixer orbital shaker (Eppendorf Iberica) at 37 °C, 1000 rpm for 30 min. Finally, reactions were stopped by placing the tubes in a water bath at 100 °C for 5 min. Supernatants were collected by centrifugation at 12,000× *g* for 5 min, and stored at −20 °C until glucose quantification. The glucose concentration of reaction mixtures was measured using the Amplex^®^ Red glucose/glucose oxidase assay kit (Invitrogen, Madrid, Spain). Absorbance was measured using a Synergy HT plate reader (Biotek) at 560 nm. Glucose concentration was calculated using a linear standard curve (0–200 μM) from a freshly prepared 400 mM stock solution. Percent inhibition was calculated relative to the negative control with 100% enzyme activity. IC_50_ values (concentration of sample in mg/mL that inhibits by 50% the original α-glucosidase activity) were calculated plotting the non-linear regression sigmoidal dose-response curves in GraphPad Prism 6.00 (GraphPad Software Inc.).

### 3.5. Separation of DPP-IV Inhibitory Peptides by Semi-Preparative RP-HPLC

Peptide purification was performed by semi-preparative RP-HPLC. Separation of peptides was performed on an HPLC system (Waters Corp., Mildford, MA, USA) equipped with two pumps (module Delta 600), a pump controller (module 600), an autosampler (module 717 plus), and a diode array detector (module 996). The data-processing software was Empower 2 (Waters Corp.). A 250 × 21.5 mm Hi-Pore 318 reverse phase column (BioRad, Hercules, CA, USA) was used. Peptide fractions were dissolved in solvent A (water: trifluoroacetic acid (TFA), 1000:1, *v/v*) at a concentration of 30 mg/mL. Fractions (400 μL) were injected and eluted at a flow rate of 10 mL/min, with a linear gradient of solvent B (acetonitrile:TFA, 1000:0.8, *v/v*) in A, going from 5% to 45% B in 50 min. Each chromatographic run was repeated 15–20 times and the subfractions were collected automatically with a Fraction Collector (Model II, Waters Corp.). The collection times were from min 6 to 12 for F1, min 12 to 16 for F2, min 16 to 22 min for F3, and min 22 to 35 for F4. The collected fractions were pooled, freeze-dried, and stored at −20 °C until further analysis. Quantification of peptides in each subfraction was performed by the Quantitative Colorimetric Peptide Assay, according to the manufacturer’s protocol.

### 3.6. Identification of Anti-Diabetic Peptides by RP-HPLC-MS/MS

RP-HPLC coupled to tandem mass spectrometry (RP-HPLC-MS/MS) analysis of collected fractions was performed on an Agilent 1100 HPLC System (Agilent Technologies, Waldbron, Germany) connected on-line to an Esquire 3000 ion trap (Bruker Daltonik GmbH, Bremen, Germany), and equipped with an electrospray ionization source. The ChemStation software (Agilent Technologies) was used for data acquisition. The column used was a reverse phase Mediterranea Sea C18 Column (150 × 2.1 mm i.d., 5 µm particle size) (Teknokroma, Barcelona, Spain). Samples were injected at 2 mg protein/mL, and peptides were eluted with a linear gradient of solvent B (acetonitrile:TFA 1000:0.27 *v/v*) in A (water:TFA 1000:0.37 *v/v*) going from 0% in 45% in 65 min at a flow rate of 0.2 mL/min. The injection volume was 100 µL. Using Data Analysis™ (version 4.0; Bruker Daltonics), the *m/z* spectral data were processed and transformed to representing mass values. The acquired MS/MS spectra were interpreted using BioTools (version 3.1; Bruker Daltonics). Peptide structures and physicochemical properties were predicted using the PepDraw tool (http://www.tulane.edu/~biochem/WW/PepDraw/). The potential biological activity of the peptides was predicted by using BIOPEP database [32].

### 3.7. Statistical Analysis

The experiments were performed with at least three independent replicates. Data are expressed as the mean ± standard deviation. Data were subjected to one-way analysis of variance (ANOVA) to compare experimental values using GraphPad Prism 6.0 software (GraphPad Software Inc.). Comparison between groups was performed using a Holm-Sidack’s test, and differences were considered significant at *p* ≤ 0.05.

## 4. Conclusions

In conclusion, this study is the first report on the ability of peptides generated from gastrointestinal digestion of germinated soybean proteins as inhibitors of DPP-IV, in addition to α-amylase and intestinal α-glucosidases. The results obtained in this work demonstrated that peptide fractions of 5–10 kDa isolated from 6DSPD have potential anti-diabetic properties. Subfractions F1, F2, and F3 showed the diversity of peptide sequences with potential candidates to inhibit DPP-IV, α-amylase, and intestinal α-glucosidases. The soybean peptide sequences identified herein should be considered for future research focusing on its ability to control hyperglycemia in preclinical animal studies and human trials. The differential composition and abundance of potential anti-diabetic peptides present in germinated soybean protein provide an extensive reservoir of bioactive sequences with potential application as ingredients or supplements in the diet to control postprandial glycemia and prevent T2DM. 

## Figures and Tables

**Table 1:** Inhibitory activity (expressed as IC_50_ or peptide concentration needed to inhibit by 50% the original enzyme activity) of six days germinated soybean protein digest (6GSPD) and peptide fractions obtained by ultrafiltration against dipeptidyl peptidase-IV (DPP-IV), α-amylase, and α-glucosidases.

	Enzymatic Inhibitory Activity (IC_50_, mg/mL) *			
Samples	DPP-IV	α-Amylase	α-Glucosidase	
			Maltase Activity	Sucrase Activity
Positive control ^1^	0.003 ± 0.000 ^a^	0.16 ± 0.01 ^a^	0.07 ± 0.01 ^a^	0.03 ± 0.00 ^a^
6GSPD	1.49 ± 0.14 ^c^	1.70 ± 0.18 ^b^	3.73 ± 0.06 ^c^	2.90 ± 0.07 ^d^
>10 kDa	1.18 ± 0.15 ^b^	4.80 ± 0.87 ^c^	>10.00 ^d^	5.27 ± 0.16 ^e^
5–10 kDa	0.91 ± 0.17 ^b^	>10.00	3.56 ± 0.14 ^c^	2.20 ± 0.40 ^c^
<5 kDa	2.21 ± 0.15 ^d^	8.30 ± 1.65 ^d^	2.56 ± 0.03 ^b^	1.23 ± 0.19 ^b^

* Values are the mean ± standard deviation of three independent experiments. Different lowercase letters indicate significant differences among samples (*p* ≤ 0.05, Holm-Sidack’s test). ^1^ Positive control was Diprotin in DPP-IV inhibitory assay and Acarbose in α-amylase and α-glucosidase inhibitory assays.

**Table 2:** Peptides identified by HPLC-MS/MS in the subfractions F1, F2, and F3 collected by RP-HPLC from 5–10 kDa fraction obtained from germinated soybean protein digest (6GSPD).

Peptide Fraction	Mass	Protein Fragment	Peptide Sequence	Net Charge ^c^	Hydrophobicity (kcal/mol) ^c^	DPP-IV Inhibitory Peptides ^a^	Antioxidant Peptides ^a^	ACE Inhibitory Peptides ^a^
**F1 ^b^**	834.3	β-conglycinin α, α’and β-chain f(270–276)	NNDDRDS	−2	+22.79	DR, ND, NN	---	---
	1013.4	β-conglycinin α and α’-chain f(295–303)	VVNPDNNEN	−2	+17.79	DN, NE, NN, NP, VN, VV	NEN	VNP
	949.4	β-conglycinin α and β-chain f(324–332)	LSSTEAQQS	−1	+13.95	QQ, QS, TE	---	EA, ST, TE
	958.4	β-conglycinin α, α’and β-chain f(530–537)	NAENNQRN	0	+18.01	AE, NA, NN, NQ, RN	---	---
	777.4	β-conglycinin α’-chain f(604–610)	IKSQSES	0	+15.36	ES, KS, QS	---	---
	981.4	Glycinin G1 subunit f(112–119)	EEPQQPQQ	−2	+18.52	EP, QP, PQ, QQ	---	PQ
	874.4	Glycinin G1 subunit f(121–128)	GQSSRPQD	0	+17.10	GQ, PQ, QD, QS, RP	---	GQ, PQ, RP
	887.4	Glycinin G2 and G1 subunit f(181–188)	LAGNQEQE	−2	+17.95	AG, LA, NQ, QE	---	AG, LA
	787.4	Glycinin G1 subunit f(465–471)	NLKSQQA	+1	+12.80	KS, NL, QA, QQ	LK	---
	972.4	Glycinin G2 subunit f(109–116)	QEPQESQQ	−2	+18.84	EP, ES, PQ, QE, QQ	---	PQ
	1150.6	Glycinin G2 subunit f(120–128)	SQRPQDRHQ	+1	+20.40	DR, PQ, QD, RH, RP	RHQ	PQ, RP
	1202.5	Glycinin G2 subunit f(193–203)	QQQQQGGSQSQ	0	+16.50	GG, QG, QQ, QS	---	GG, GS, QG
	1387.7	Glycinin G2 subunit f(193–205)	QQQQQGGSQSQKG	+1	+20.46	GG, KG, QG, QQ, QS	---	GG, GS, KG, QG, QK
	1244.6	Glycinin G2 and G5 subunit f(198–207)	PETMQQQQQQ	−1	+15.87	ET, MQ, QQ, TM	---	---
	1311.5	P34 Probable thiol protease f(250–261)	SDESTESETEQA	−5	+29.21	ES, ET, QA, TE	---	ST, TE
**F2**	1546.7	Glycinin G2 subunit f(238–251)	RNLQGENEEEDSGA	−4	+32.34	GA, GE, NE, NL, QG, RN	---	GA, GE, LQ, QG, SG
	843.5	Glycinin G4 subunit f(451–458)	VTRGQGKV	+2	+14.89	GQM KVM QG, RG, TR, VT	---	GK, GQ, QG, RG, GKV, VTR
	644.4	P34 Probable thiol protease f(143–148)	KKGVIT	+2	+13.32	GV, KG, KK, VI	---	GV, KG
	1555.6	P34 Probable thiol protease f(248–261)	IMSDESTESETEQA	−5	+27.42	ES, ET, IM, QA, TE	---	ST, TE
**F3**	1497.7	Glycinin G1 subunit f(37–50)	NALKPDNRIESEGG	−1	+26.14	AL, DN, EG, ES, GG, KP, NA, NR, RI	LK, KP, LKP	EG, GG, IE, KP, LKP
	1433.7	Glycinin G1 subunit f(329–342)	SSPDIYNPQAGSVT	−1	+14.43	AG, NP, PQ, QA, SP, SV, VT, YN	IY	AG, GS, IY, PQ, YN, AGS
	1497.7	Glycinin G2 subunit f(34–47)	NALKPDNRIESEGG	−1	+26.14	AL, DN, EG, ES, GG, KP, NA, NR, RI	LK, KP, LKP	EG, GG, IE, KP, LKP
	1956.9	Glycinin G2 subunit f(312–329)	RQNIGQNSSPDIYNPQAG	0	+19.57	AG, GQ, PQ, QA, QN, SP, YN	IY	AG, GQ, IG, IY, PQ, YN
	1497.7	Glycinin G3 subunit f(37–50)	NALKPDNRIESEGG	−1	+26.14	AL, DN, EG, ES, GG, KP, NA, NR, RI	LK, KP, LKP	EG, GG, IE, KP, LKP
	1261.6	Glycinin G4 subunit f(486–497)	VVAEQAGEQGFE	−3	+21.00	AE, AG, GE, GF, QA, QG, VA, VV	---	AG, GE, GF, QG
	882.5	β-conglycinin α-chain f(190–196)β-conglycinin α’-chain f(206–212)	HKNKNPF	+2	+15.96	NP, PF	---	HK, NK

^a^ Data accessed from BIOPEP database, available at http://www.uwm.edu.pl/biochemia/index.php/pl/biopep on July 2018; ^b^ Mass, peptide sequence and protein fragment of F1 were reported previously in Gonzalez-Montoya et al. [9]; ^c^ Net charge (sum of positively (basic) and negatively (acidic) charged residues at neutral pH) and hydrophobicity (Wimley-White scale) of peptide sequences were predicted using PepDraw tool at http://www.tulane.edu/~biochem/WW/PepDraw/.

## Abbreviations

6GSPD	6-day germinated soybean protein digest
DM	diabetes mellitus
DPP-IV	dipeptidyl peptidase IV
MGAM	maltase-glucoamylase
RP-HPLC-MS/MS	RP-HPLC coupled to tandem mass spectrometry
SI	sucrase-isomaltase
T2DM TFA	type 2 diabetes mellitus trifluoroacetic acid
